# Understand the Potential Role of *Aureobasidium pullulans*, a Resident Microorganism From Grapevine, to Prevent the Infection Caused by *Diplodia seriata*

**DOI:** 10.3389/fmicb.2018.03047

**Published:** 2018-12-11

**Authors:** Cátia Pinto, Valéria Custódio, Mariana Nunes, Aurélie Songy, Fanja Rabenoelina, Barbara Courteaux, Christophe Clément, Ana Catarina Gomes, Florence Fontaine

**Affiliations:** ^1^SFR Condorcet – FR CNRS 3417, Unité Résistance Induite et Bioprotection des Plantes (RIBP), Université de Reims Champagne-Ardenne, Reims, France; ^2^Genomics Unit, Biocant – Biotechnology Innovation Center, Cantanhede, Portugal; ^3^Center for Neurosciences and Cell Biology, University of Coimbra, Coimbra, Portugal

**Keywords:** *Aureobasidium pullulans* strain Fito_F278, GTD, *Diplodia seriata*, plant–microbial interaction, antagonistic potential, grapevine colonization, grapevine responses

## Abstract

Grapevine trunk diseases (GTDs) are one of the major concern amongst grapevine diseases, responsible for the decline of vineyards and for several economical losses. Since grapevine is naturally colonized by resident microorganisms such as *Aureobasidium pullulans*, the present challenge is to understand their biocontrol potential and how such microorganisms can be successfully integrated in the control of GTDs. In this context, the first priority consists to exploit the plant-beneficial-phytopathogen interactions in plant model systems, to identify the most prevalent equilibrium limiting expression of GTDs. In the current study, we deep characterized the interaction of a resident and abundant microorganism from grapevine – *Aureobasidium pullulans* strain Fito_F278 – against *D. seriata* F98.1, a Botryosphaeria dieback agent, and with plant (cv Chardonnay). Results revealed that *A. pullulans* strain Fito_F278 was able to reduce significantly the mycelium growth of *D. seriata* F98.1 at 33.41 ± 0.55%, under *in vitro conditions*, though this reduction is possibly dependent on a direct interaction between strain Fito_F278 and pathogen. Furthermore, strain Fito_F278 was able to promote an induction of some plant defense responses in cutting plants, 1 week after the *D. seriata* F98.1 infection. Results evidenced that strain Fito_F278 colonized efficiently grapevine at both epiphyte and endophyte level, could persist on plant roots for long-periods (up to 2 months after its inoculation) and grow at different pH and high salinity conditions. Moreover, a significant decrease of the microbial load from soil and rhizosphere was observed in plants treated with the strain Fito_F278, suggesting its competitivity potential in a microbial ecosystem. Altogether, the present study gives the first insights about the interaction of *A. pullulans* strain Fito_F278, a resident microorganism, with grapevine, its potential role against a Botryosphaeria dieback agent, and highlights its importance to toward more resilient grapevine.

## Introduction

Grapevine is largely attacked by different phytopathogens such as those responsible for powdery mildew, downy mildew, and gray mold (Armijo et al., 2016). However, those causing grapevine trunk diseases (GTDs) such as Esca disease, Eutypa and Botryosphaeria diebacks, are of utmost concern to wine industry as these are the most destructive grapevine diseases worldwide (Mugnai et al., 1999; Larignon et al., 2009; Bertsch et al., 2012; Fontaine et al., 2016a). Among GTDs, the most frequent is Esca, a disease complex, in which different fungal pathogens are associated and that include *Phaeomoniella chlamydospora, Phaeoacremonium minimum*, and *Fomitiporia mediterranea*. Moreover, *Eutypa lata* and *Stereum hirsutum* may be also involved (Mugnai et al., 1999; Bertsch et al., 2012). Eutypa dieback is caused by *Eutypa lata* although *Eutypa leptoplaca, Cryptovalsa ampelina, Diatrypella* spp. or *Eutypella* spp. could be present (Fontaine et al., 2016b; Belda et al., 2017). Finally, Botryosphaeria dieback is caused by *Botryosphaeriaceae* species such as *Diplodia seriata, Botryosphaeria dothidea, Diplodia mutila*, or *Neofusicoccum parvum* and are the causal agents of trunk cankers and grapevine decline (Larignon et al., 2009; Úrbez-Torres, 2011; Fontaine et al., 2016a,b).

So far, no efficient treatments are available to limit emergence of GTDs, which constitutes a global threat to wine heritage and with negative repercussions at social and economic levels (Bertsch et al., 2012; Fontaine et al., 2016a). In this context, sustainable alternatives to prevent GTDs have been discussed and proposed, and it was reported that more than 40 potential BCAs have been tested against GTDs (Mondello et al., 2018). Interestingly, the most studied have been *Trichoderma* spp. and *Bacillus* species, which were tested against the three main GTDs, albeit *Streptomyces* spp., different *Pseudomonas* species and the yeast *A. pullulans* were also tested for their biocontrol against Eutypa dieback (Mondello et al., 2018).

In the last years, the characterization of the grapevine microbiome has been the object of study (reviewed in Pinto and Gomes, 2016; Belda et al., 2017). Considering that grapevine represents a natural reservoir of resident microbial resources embedded in a complex micro-ecosystem (Pinto and Gomes, 2016), the better knowledge of these communities and their interactions with grapevine will allow both identification and characterization of new microorganisms with biocontrol potential from and for grapevine protection and, to promote advances in management of GTDs. Among this microbial ecosystem, *Aureobasidium pullulans* is known to dominate the microbial consortia of grapevine and is recognized by its high range distribution over plant, both at below- and above-ground parts (Sabate et al., 2002; Martini et al., 2009; Grube et al., 2011; Barata et al., 2012; Pinto et al., 2014) although its functional role has not been fully elucidated. In this context, the present study aims to analyze *Aureobasidium pullulans* strain Fito_F278, isolated from grapevine leaves, (i) to better understand its patterns of grapevine colonization, and (ii) to evaluate its efficacy to protect grapevine against the spread of Botryosphaeria dieback pathogen, namely *Diplodia seriata* F98.1.

## Materials and Methods

An experimental design scheme representing all the approaches under this study is represented in Supplementary Figure S1.

### Microbial Strains and Growth Conditions

Two strains of *Diplodia seriata* (strains F98.1 (Robert-Siegwald et al., 2017) and Ds99.7) associated with Botryosphaeria dieback were selected for this study. Strain F98.1 was isolated from *Vitis vinifera* cv Syrah (Pyrénées – Orientales, France) and strain Ds99.7 was isolated from *V. vinifera* cv Clairette (Rhône-Alpes, France). These phytopathogens were selected based on their patterns of aggressiveness, ranging from high (Ds99.7) to low virulence (F98.1) (Ramírez-Suero et al., 2014; Reis et al., 2016). Stock cultures were maintained on Potato Dextrose Agar (PDA) at 4°C. For inoculation tests, each corresponding strain was cultured in Petri dishes containing PDA medium for 7 days at 28°C.

*Aureobasidium pullulans* strain Fito_F278 was isolated from leaves of *V. vinifera* in 2011 at Bairrada appellation in Portugal. This isolate belongs to a microbial collection of Genomics Unit from Biocant (Portugal) and the stock culture was maintained in 80% (v/v) glycerol at -80°C. The molecular identification of strain Fito_F278 was performed by sequencing the ITS region using the 3500 Genetic Analyser (Applied Biosystems) at Biocant, Portugal. Sequence was then queried by blast on nt@ncbi for taxonomic annotation and is publicly available on GenBank with the accession number MF983874. For the *in vitro* screening of the antagonistic activity, strain Fito_F278 was grown on YPD agar for 48 h at 28°C. Regarding the *in vivo* experiments for the analysis of plant colonization and biocontrol activity, fresh strain cells were obtained on YPD broth and collected by centrifugation at 4,500 rpm for 10 min at 4°C and washed twice with sterile PBS pH 7.5 (NaCl 8 g/L; KCl 0.2 g/L; Na_2_HPO_4_ 1.44 g/L; KH_2_PO_4_ 0.24 g/L). Cell concentration was then adjusted to 10^6^ CFU/mL for plantlet colonization assay and to 10^7^ CFU⋅g^-1^ of soil for the greenhouse bioassay.

### *In vitro* Assessment of Antifungal Effects

#### Screening of the Antagonistic Activity in Dual Culture

A dual culture screening was performed to test the antagonistic potential of strain Fito_F278 on the mycelial growth of *D. seriata* (Supplementary Figure S1a). For this, a mycelial plug of the phytopathogen (3 mm of diameter) aged of 7 days old was placed at 1 cm from the border of a Petri dish containing PDA medium and a loop of Fito_F278 colonies was placed at the opposite side. Plates inoculated only with the phytopathogen served as control. The assay was performed in triplicate and plates were incubated in the dark at 28°C and followed for 15 days. The inhibitory effect of each isolate was calculated based on (i) the relative mycelium inhibition (MI) through the formula MI% = ((Mfg-Mga)/Mfg) × 100, where Mfg corresponds to the diameter of the free mycelium growth of the phytopathogen (control) and Mga to the diameter of the mycelium growth of the phytopathogen in the presence of the antagonistic microorganism (dual culture); (ii) and through the area of phytopathogen mycelium growth by using Image J 1.50b software (National Institutes of Health, United States).

#### Mechanisms Behind the Antagonistic Activity of Strain Fito_F278

Strain Fito_F278 was further investigated for the mechanisms behind its antagonistic potential, namely screening for the presence of siderophores, phosphate solubilization and enzymatic activity (Supplementary Figure S1a). The enzymatic activity was analyzed for amylase, cellulase, lipase, pectinase, protease, and urease production.

##### Presence of siderophores

The method of Chrome Azurol S (CAS) described by Alexander and Zuberer (1991) was used to detect the presence of siderophores, through the yellow halo formation around colonies.

##### Phosphate solubilization

The phosphate solubilization was analyzed with Pikovskaya culture medium [Glucose 10 g/L; NaCl 0.2 g/L; (NH_4_)_2_(SO_4_) 0.5 g/L; Yeast extract 0.5 g/L; MnSO_4_ 0.1 g/L; MgSO_4_ 0.1 g/L; Agar 20 g/L and Ca_3_(PO_4_) 5 g/L that was sterilized separately] and the degradation halo (clear zone) around colony corresponded to a positive activity. For both tests, plates were incubated until a period of 10 days at 28°C and tests were performed in triplicate.

##### Amylase

For the amylolytic activity, the strain was drop-spotted onto PDA (Merck) for 48 h at 28°C which was then flooded with 5 mL iodine solution for 2 min.

##### Cellulase

The cellulase production was assessed according to Kasana et al. (2008). For this, Fito_F278 was drop-spotted onto CMC agar [NaNO_3_ 2 g/L; K_2_HPO_4_ 1 g/L; MgSO_4_ 0.5 g/L; KCl 0.5 g/L; carboxymethylcellulose (CMC) sodium salt 2 g/L; peptone 0.2 g/L; agar 17 g/L] for 48 h at 28°C and then flooded with 5 mL of iodine solution for 2 min.

##### Lipase

The lipase production was confirmed after drop-spotting the strain onto PDA supplemented with 1% Tween-20 (Hasan et al., 2013), a lipid substrate, and incubated for 48 h at 28°C.

##### Pectinase

The capacity to hydrolyze pectin was assessed by drop-spotting the Fito_F278 on nutrient agar (NA) (peptone 5 g/L; beef extract 3 g/L; NaCl 5 g/L; Agar 15 g/L; pH 6.8) supplemented with 0.2% of pectin, incubation for 48 h at 28°C and then flooded with 5 mL of iodine solution for 2 min.

##### Protease

The proteolytic activity was confirmed according to Hasan et al. (2013). Fito_F278 was inoculated in Petri dishes with NA supplemented with 1% of gelatin, a protein source, and incubated for 48 h at 28°C.

##### Urease

The urease screening was detected according to Seeliger (1956), with some modifications. The Christensen’s culture media (peptone 1 g/L; glucose 1 g/L; NaCl g/L; KH_2_PO_4_ 2 g/L; phenol red 0.012 per 1 L; agar 20 g/L; pH 6.8) was distributed in 1.5 mL microtubes and a drop of 20% urea solution, sterilized by filtration, was added. The strain was then inoculated and incubated until a period of 5 days at 28°C. The urea hydrolysis causes a color change of the media from orange-yellow to pinkish red.

Overall, results were expressed by either positive activity, when a clear halo around strain colony was observed, or negative activity. The enzymatic index (EI) was calculated by the relationship between the average diameter of the degradation halo (cm) and the average diameter of the colony growth (cm). All enzymatic activity tests were performed in triplicate, and for each plate, strain Fito_F278 was inoculated twice. The negative control consisted of a Petri dish containing the specific culture media without strain inoculation.

#### Physiological Activity of Strain Fito_F278

The capacity of strain Fito_F278 to survive under harsh environmental conditions was analyzed for two physiological traits, such as pH and salinity conditions (Supplementary Figure S1a). For pH analysis, the strain was streaked in YPD medium and adjusted with pH 5, 6, 7, 9, and 11 and incubated for 48 h at 28°C. For the salinity effect, the strain was streaked in YPD medium adjusted with different salt concentrations (0, 2, 4, 6, 8, 10, 12, and 14% of NaCl), and incubated for 72 h at 28°C. Experiments were performed in triplicate.

### *In vivo* Assays for Colonization and Biocontrol Activity Assessment

#### Efficacy of Strain Fito_F278 to Colonize Grapevine Plantlets

##### Experimental design

Microbe-free plantlets of *V. vinifera* L. cv Chardonnay clone 7535 were propagated by nodal explants in culture tubes with 25 mm diameter, containing 15 mL of Martin Medium (Dean et al., 2012). Plants were grown in a growth chamber under white fluorescent light (200 μmol⋅m^-2^⋅s^-1^), 16 h photoperiod and at a temperature constant of 26°C (Compant et al., 2005a). Plantlets with 5-week-old were then selected and for each experiment, two conditions were performed, namely (i) control (non-inoculated plants) and (ii) plants inoculated with strain Fito_F278. Each condition contained *n* = 15 similar plants and the experiment was repeated twice.

Plant inoculation was performed by dipping the plant roots in a 5 mL of fresh strain suspension at 10^6^ CFU/mL in PBS pH 7.5 or PBS pH 7.5 (control) for 10 s. Plantlets were then carefully transferred to a Magenta box containing 100 mL of semi-solid Martin Medium (Dean et al., 2012) and incubated in the growth chamber as described above (Supplementary Figure S1b). Each Magenta Box contained 2 plantlets.

##### Plant colonization analysis

Results were evaluated at 4, 7- and 14-days’ post root inoculation (dpi) (Supplementary Figure S1b). For each sampling time and condition, roots and leaves of 5 plantlets were selected, pooled together and 2 biological replicates were performed. Samples were weighted and rinsed in sterile distilled water, analyzed by classic microbiology through plate counting by molecular biology and observed using a three-dimensional (3D) microscope (VHX-2000 series) to check any possible impact of strain colonization on plant physiology. Both epiphytic and endophytic patterns of plant colonization were achieved. For epiphytic colonization, samples were ground in 1 mL of PBS pH 7.5 followed by 10-fold serially dilutions and plating of 100 μL. For endophytic colonization, samples were surface sterilized with 70% ethanol for 1 min, followed by 0.6% (v/v) sodium hypochlorite for 3 min and washed four times in distilled water. Samples were then ground in 1 mL of PBS pH 7.5, 10-fold serially diluted and plated. To ensure the efficacy of the sterilization step, 100 μL of the last wash solution of each condition was also cultured. For both analysis, colonies were counted after 48–72 h of incubation at 28°C. The identification of strain Fito_F278 was confirmed by using strain-specific primers targeting the Glutathione S-transferase C (GST) gene (MK130989) and by sequencing the ITS region. Primer sequences were: GST_F (5′-GCTGACCGCAATTCGCATAC-3′) and GST_EF1R (5′-GTTGCTCATGAAGGTGAGGG-3′); ITS1 (5′-TCCGTAGGTGAACCTGCGG-3′) and ITS4 (5′-TCCTCCGCTTATTGATATGC-3′) (White et al., 1990). The genomic DNA of roots and leaves was extracted using the QIAamp^®^ DNA Stool Mini Kit (Qiagen), while genomic DNA from cell colonies was extracted using the Wizard Genomic DNA Purification Kit (Promega). Distinct PCR reactions were performed for GST and ITS region. PCR reactions for GST region were carried out in 25 μL reaction mix containing 1x Dream Taq buffer with MgCl_2_ (Thermo Scientific), 0.2 mM dNTPs ((Thermo Scientific), 0.2 μM of each primer, 1.25 U of Dream Taq DNA polymerase (Thermo Scientific) and 2 μL of genomic DN. Cycling conditions consisted in a first denaturation step at 94°C for 4 min followed by 30 cycles at 94°C for 30 s, 58°C for 30 s, and 72°C for 45 s. A final extension cycle at 72°C for 5 min was applied. The final product is a 753 bp fragment that indicates the presence of strain Fito_F278 DNA in the samples analyzed. The ITS reactions contained 1x reaction buffer, 2 mM MgCl_2_, 0.2 mM dNTPs (Bioron), 1 U of Taq DNA Polymerase, 0.4 μM of forward and reverse primers and 2 μL of genomic DNA. Cycling conditions consisted in a first denaturation step at 95°C for 6 min followed by 35 cycles at 94°C for 40 s, 53°C for 40 s, and 72°C for 1 min, and a final extension cycle at 72°C for 5 min.

#### Efficacy of Strain Fito_F278 to Protect Grapevine Against Diplodia Seriata F98.1

##### Experimental design

The ability of strain Fito_F278 to protect cv. Chardonnay cuttings aged of 8 weeks following the artificial inoculation of *D. seriata* F98.1 was tested under greenhouse conditions. Before planting, cuttings were twice disinfected with 0.05% cryptonol (8-hydroxyquinoline sulfate) overnight to minimize or even to avoid the presence of any microorganism.

The experimental conditions consisted of (i) non-inoculated plants (control), (ii) plants inoculated with strain Fito_F278, (iii) plants inoculated with the phytopathogen F98.1 and (iv) plants inoculated with both F98.1 + Fito_F278. The phytopathogen was artificially inoculated on green stems in the second internode, while strain Fito_F278 was inoculated on soil (Supplementary Figure S1c). For the experimental conditions (ii) and (iv), cuttings were inoculated twice with a 30 ml of fresh strain Fito_F278 solution at 10^7^ CFU⋅g^-1^ of soil (namely at T1 and T2), while the control condition (i) was inoculated with PBS pH7.5. These treatments were performed with a week of interval. Three weeks after the second treatment (T2), a plug containing the phytopathogen or a PDA plug (control condition) was individually inoculated in green stems (T3), according to the model described by Spagnolo et al. (2017). Each condition contained a total of 10 plant replicates and the experiment was repeated twice.

##### Evaluation of green stem necrosis and re-isolation of *D. seriata* F98.1 and strain Fito_F278

Four weeks after the phytopathogen inoculation (T3+4W), plants of each treatment condition were used for visual evaluation of the external necrosis of green stems, and for the re-isolation of *D. seriata* F98.1 (Supplementary Figure S1c). The external lesions of cutting wood were measured for their width and length from the wound inoculation, and the re-isolation process was carried out from green stems at the artificial inoculation spot (necrotic tissues) and at 1 cm both above and below, according to the protocol of Larignon and Dubos (1997), that was adapted. For this, wood tissues were rapidly passed over a flame, the top of the necrotic zone removed with a scalpel and six small tissue pieces per plant were plated onto malt extract agar (MEA, 20 g/L) supplemented with 0.015% of sulfate streptomycin. Plates were then incubated for a minimal of 7 days and a maximal of 15 days at 24°C. Isolations were performed from 4 plants in each experiment. Results were expressed as a relative frequency (%) of necrotic pieces infected.

The re-isolation of *A. pullulans* strain Fito_F278 was carried out from soil, rhizosphere, roots and leaves at T3+1 week and T3+4 weeks (Supplementary Figure S1c). 0.1 g of soil or root were weighted, and 0.9 mL of sterile distilled water added and vortexed, followed by a 10-fold serial dilutions and plating of 100 μL in YPD. Root and leaves were analyzed only for the endophytic colonization and according to the methodology described above in the plant colonization analysis section. The presence of strain Fito_F278 was confirmed by using strain-specific primers targeting the GST and by sequencing the ITS region. Herein, a nested-PCR reaction was performed for the GST gene, while the ITS region was analyzed as previously described in the plant colonization analysis section. The master mix for the first PCR consisted in a 25 μL reaction mix containing 1x Dream Taq buffer with MgCl_2_ (Thermo Scientific), 0.5 mM MgCl_2_ (Thermo Scientific), 0.2 mM dNTPs (Thermo Scientific), 0.2 μM of each primer (GST_F and GST_EF1R), 1.25 U of Dream Taq DNA polymerase (Thermo Scientific) and 0.5 μL of genomic DNA. The mixture was incubated at 94 °C for 4 min; 20 cycles of 94°C for 30 s, 58°C for 30 s, and 72°C for 45 s; and a final extension at 72°C for 5 min. For the second (nested) PCR, the mix was similar to the first, except that 0.5 μL of the first PCR product was used as template DNA, the primers used were GST_F2 (5′-CTGTCGGTGCCCTTGAGGA-3′) and GST_EF1R1 (5′-CGTCGTTGTACTTGTAGTCC-3′), and the reaction mixture was incubated at 94°C for 4 min; 30 cycles of 94°C for 30 s, 57°C for 30 s, and 72°C for 45 s; and a final extension at 72°C for 5 min. The final product is a 516 bp fragment that indicates the presence of strain Fito_F278 DNA in the samples analyzed.

##### Evaluation of chlorophyll *a* fluorescence

Measurements of the chlorophyll *a* fluorescence levels, namely the activity of the photosystem II (PSII), a sensitive marker of the plant’s early responses to stresses (Chapin et al., 1993; Letousey et al., 2010), was quantified by using the pulse modulated chlorophyll fluorometer system (FMS2, Hansatech instruments). The optimal quantum yield of PSII electron transport (φPSII) was automatically calculated by the formula *([Fm – F0]/Fm*), where *F0* is the minimal fluorescence and *Fm* the maximal fluorescence (Genty et al., 1989). This indicated the amount of light absorbed by the chlorophyll *a* associated with the PSII. Thus, a decline of φPSII may be associated with a down-regulation of the electron transport (Nogués et al., 2002; Petit et al., 2006). Measures were performed in all plants at the first (L1) and forth (L4) foliar level above the artificial inoculation spot of the phytopathogen and at T3, T3+3 days, T3+1 week, T3+2 weeks, T3+3 weeks and T3+4 weeks (Supplementary Figure S1c).

##### Plant total RNA extraction and Real-time PCR (qPCR) analysis of gene expression

Green stems and leaves at the first (L1) and fourth (L4) foliar level were sampled above the artificial inoculation spot of the phytopathogen, and immediately frozen in liquid nitrogen and stored at -80°C. Leaves were collected at 3 days (T3+3 days) and 1 week (T3+1W) after the phytopathogen inoculation, while green stems were collected only at 1 week (T3+1W) (Supplementary Figure S1c). Samples (pooled of leaves and/or green stems from four plants) were ground in liquid nitrogen to a fine powder. Total RNA extraction was carried out from 50 mg of powdered leaves or green stems, and by using the Plant RNA Purification Reagent (Invitrogen, France). Given the green stems, two independent RNA extractions were performed for each sample, which were then pooled after extraction. The RNA pellet was re-suspended in 15 μL (leaves) or 8 μL (green stems) of RNase-free water and treated with RD1 DNase enzyme (Promega Corp.), to avoid any genomic DNA contamination. The RNA integrity was checked by agarose gel electrophoresis and the quantity determined by measuring the absorbance at 260 nm.

The reverse transcription was carried out on 150 ng of total RNA using the Verso cDNA synthesis kit (Thermo Fisher Scientific), and the cDNAs were then stored at -20°C. The 8 targeted genes (Table 1) were selected from previous studies focused on grapevine responses to GTDs (Magnin-Robert et al., 2011; Reis et al., 2016; Spagnolo et al., 2017). These genes included: 2 genes encoding for pathogenesis-related (PR) proteins: PR protein 6 (*PR6*) and β-1,3 glucanase (*Gluc*); 3 genes involved in detoxification and stress tolerance: haloacid dehalogenase hydrolase (*HahL*), glutathione S-transferase (*GST5*) and α-crystalline heat shock protein (*HSP*); 1 gene involved in the phenylpropanoid pathway: stilbene synthase (*STS*); 1 gene involved in the cellwall compound synthesis: fasciclin-like arabinogalactan protein (*fascAGP*); and 1 gene encoding for an aquaporin plasma membrane intrinsic protein 2-2 (*PIP2.2*) (Table 1). The α-chain elongation factor 1 gene (*EF1-α*) and 39S ribosomal protein L41-A (*39SRP*) were used as reference genes for leaves samples, while the alcohol dehydrogenase 2 gene (*ADH2*) and 60S ribosomal protein L18 (*60SRP*) were used as reference genes for green stems (Table 1). These reference genes were already described in other studies using the same patho-system (Reis et al., 2016; Spagnolo et al., 2017), and were selected based on their target stability value (stability measure M) after a rank of candidate reference genes. The qPCR was then carried out by using the Absolute Blue qPCR SYBR Green ROX mix (Thermo Fisher Scientific Inc.), according to the manufacture’s protocol, and in the CFX96 thermocycler system (Bio-Rad, Hercules, CA, United States). All reactions were performed in duplicate in 96-well plates, using the following thermal conditions: 15 s at 95°C (denaturation) and 1 min at 60°C (annealing and extension) for 40 cycles. Melting curves were performed from 65 to 95°C at 0.5°C⋅s^-1^, and melting peaks allowed to check the specificity of each amplification (Supplementary Table S1). Data was analyzed with Bio-Rad CFX Manager 3.0 software (Bio-Rad), as the cycle of quantification (Cq). A mean of Cq value was obtained for each gene and sample; then ΔCq value was calculated for each sample by the difference between the ΔCq of the target and reference gene. Results of each sample were expressed as relative expression to the control condition (non-inoculated plants) corresponding to a fixed value of 1, and were calculated with the Livak method, also known as 2^-ΔΔCq^ method, where ΔΔCq corresponds to the ΔCq between two samples (inoculated sample and control sample). Results were expressed as mean and standard error values (SEM) of duplicate reactions from two independent experiments; normalized with two reference genes (Vandesompele et al., 2002). The relative expression of genes was considered up- or down- regulated when changes in their expression were > 2-fold or <0.5-fold, respectively, as indicated in previous work (Reis et al., 2016; Magnin-Robert et al., 2017).

**Table 1:** Primer sequences used for amplification of reference and target genes, amplicon length, amplification temperature (Ta) and melting temperature (Tm).

Function	Gene	Primer Sequence (forward/reverse primer)	Accession number	Matrix	Amplicon length (bp)	Melting temperature (Tm)	Amplification temperature (Ta)
Reference genes	*EF1* (EFl-α elongation factor)	5′-GAACTGGGTGCTTGATAGGC -3′/ 5′- AACCAAAATATCCGGAGTAAAAGA-3′	GU585871	Leaves	150	81.5°C	
	39SRP (39S Ribosomal protein L41-A)	5′ GACTGACTTCAAGCTTAAACC -3′/ 5′ GATATAACAGGGAATACAGCAC-3′	XM 002285709.1		282	81°C	
	*ADH2* (Alcohol dehydrogenase 2)	5′-GACCATGTTCTTCCTGTATTCAC-3′/ 5′- GTAGCACCAAGACCTGTAGAG-3′	NM_001281154.1	Green stem	293	82.5°C	
	60SRP (60S Ribosomal Protein L18)	5′-ATCTACCTCAAGCTCCTAGTC -3′/ 5′- CAATCTTGTCCTCCTTTCCT -3′	XM 002270599		166	81.5°C	
Defense proteins	*Glue* (β-1,3 glucanase)	5′-TCAATGGCTGCAATGGTGC -3′/ 5′- CGGTCGATGTTGCGAGATTTA-3′	DQ267748	Leaves/green stem	155	81°C	
	*PR6* (Serine-protease inhibitor 6)	5′-AGGGAACAATCGTTACCCAAG -3′/ 5′- CCGATGGTAGGGACACTGAT-3′	AY156047	Leaves	91	80.5°C	
Detoxification and stress tolerance	*Hahl* (Haloacid dehalogenase hydrolase)	5′-CCCTCAGGATAGCCAACATCA -3′/ 5′- AGGTGCCAACCAGAACTGTGT-3′	NM_001281217	Leaves/green stem	111	81°C	60°C
	*HSP* (alpha crystalline heat shock protein)	5′-TCGGTGGAGGATGACTTGCT -37 5′- CGTGTGCTGTACGAGCTGAAG-3′	XM_002272382	Leaves/green stem	101	81°C	
	GST5 (Glutathione S-transferase 5)	5′-GCAGAAGCTGCCAGTGAAATT-3′/ 5′- GGCAAGCCATGAAAGTGACA-3′	XM 002277883	Leaves	101	79°C	
Phenylpropanoid metabolism	*STS* (Stilbene synthase)	5′-AGGAAGCAGCATTGAAGGCTC -3′/ 5′- TGCACCAGGCATTTCTACACC-3′	FJ851185	Green stem	101	80.5°C	
Cell wall compounds	*fascAGP* (fasciclin-like arabinogalactan protein)	5′-CGAAACCCCAAAGCCTAAGAA -3′/ 5′- GAAAACACAAAGGGGTTGCA-3′	XM 002280793.2	Green stem	101	80.5°C	
Aquaporin	*PIP2.2* (aquaporin plasma membrane intrinsic protein 2-2)	5′-GGTTCAGTCTCCATTGCACATG -3′/ 5′- TTGGCAGCACAGCAGATGTAT-3′	NM 001280956	Green stem	101	78°	

### Statistical Analysis

The significance of difference of experimental data was analyzed by using an analysis of variance (ANOVA), followed by Tukey and/or Bonferroni *post hoc* analysis, and a confidence limit of 95% was applied. The assumptions of ANOVA were determined through Shapiro–Wilk test (*p* < 0.05) for normality test, and Levene’s test (*p* < 0.05) for homogeneity of variances in the residuals. When the assumptions for a parametric ANOVA were rejected, the non-parametric Kruskal-Wallis and Mann–Whitney *U* test were applied. Standard errors of the mean (SEM) were calculated for the mean values. For plant colonization analysis, the obtained CFU count was transformed to the logarithmic scale. The statistical software package used was SPSS (version 20.0, SPSS, Inc., Armonk, NY, United States), and the graphs were performed by using GraphPad Prism (version 5.01, GraphPad Software, Inc., San Diego, CA, United States).

## Results

### *In vitro* Tests

#### Evaluation of the Antagonistic Activity of *Aureobasidium pullulans* Strain Fito_F278

**FIGURE 1:**
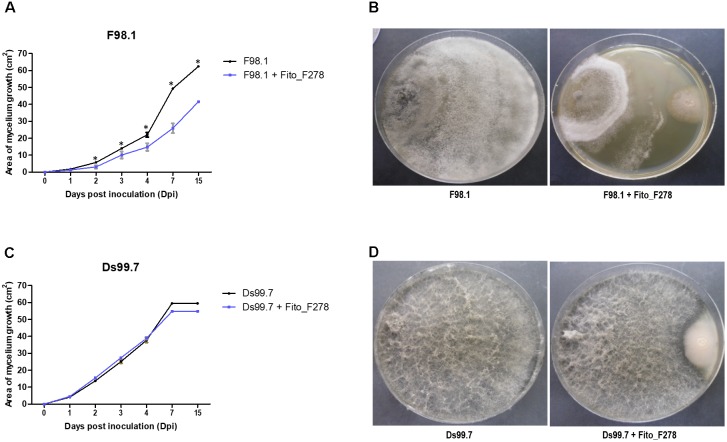
Evaluation of the antagonistic activity of *Aureobasidium pullulans* strain Fito_F278 against Botryosphaeria dieback agents. Kinetics of the mycelium growth (cm^2^) of *D. seriata* strain F98.1 **(A)** and Ds99.7 **(C)** from 0 to 14 days post-inoculation (dpi), and antagonistic activity of strain Fito_F278 against *D. seriata* F98.1 **(B)** and Ds99.7 **(D)** after 14 dpi. Results correspond to the area of phytopathogens growth of three biological replicates assessed for each condition; the experiment was carried out twice. The mean and standard error values of 3+3 replicates from two experiments are presented for each condition. The ^∗^ indicates significant differences after a pairwise comparisons between control (*D. seriata* F98.1 or Ds99.7) and *Aureobasidium pullulans* strain Fito_F278 for each time point, according to the Mann–Whitney *U* test (*p* ≤ 0.05).

The antagonistic activity of strain Fito_F278 was tested against two different strains of *D. seriata* (strains F98.1 and Ds99.7), through a dual screening on PDA. Strain Fito_F278 was able to reduce significantly the mycelium growth of *D. seriata* F98.1 (*p* < 0.05) after 14 dpi, with a mycelium inhibition of 33.41 ± 0.55% (Figures 1A,B). In contrast, no significant inhibition on the mycelium growth of *D. seriata* Ds99.7 was observed for the same period (7.98 ± 0.95%) (Figures 1C,D). For both phytopathogens, the mycelial inhibition was notable after 4 dpi.

#### Evaluation of *Aureobasidium pullulans* Strain Fito_F278 for Its Enzymatic Production and Physiological Traits

**FIGURE 2:**
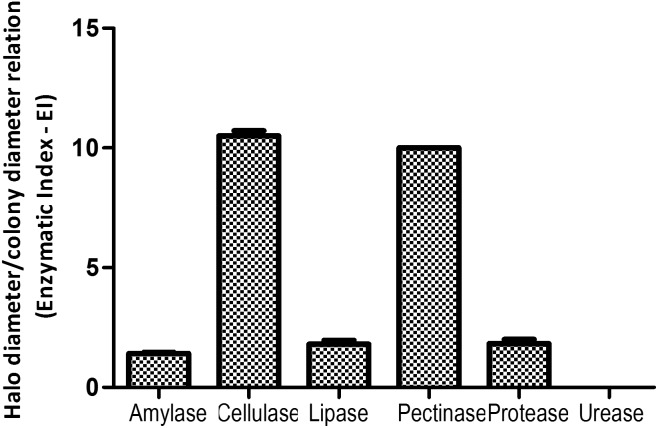
Extracellular enzymatic activity profile of *Aureobasidium pullulans* strain Fito_F278. Analysis of amylase, cellulase, lipase, pectinase, protease, and urease activities for strain Fito_F278. The enzymatic index (EI) was calculated by the relationship between the average diameter of the degradation halo (cm) and the average diameter of the colony growth (cm). Results of EI are of two biological replicates assessed for each condition and the experiment was carried out three times. The pooled mean and standard error values of 2 + 2 + 2 replicates from the three experiments are presented for each condition.

*Aureobasidium pullulans* strain Fito_F278 was able to produce siderophores and to solubilize the phosphate under *in vitro* conditions (data not shown). This strain also showed an important extracellular enzymatic activity (Figure 2), and both cellulase (10.50 ± 0.20) and pectinase (10.00 ± 0.00) showed the highest enzymatic index, followed by the protease (1.83 ± 0.15), lipase (1.81 ± 0.15) and amylase (1.42 ± 0.05) activity. No urease activity was detected under *in vitro* conditions.

**FIGURE 3:**
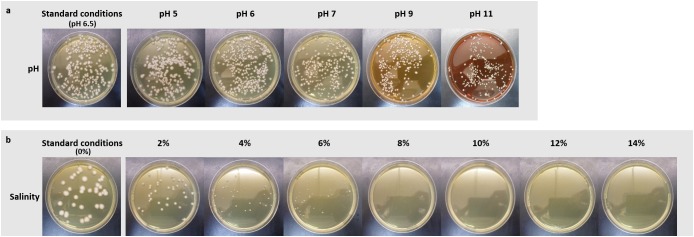
Evaluation of the physiological traits (pH and salinity) on the growth of *Aureobasidium pullulans* strain Fito_F278. Effect of pH **(a)** and salinity **(b)** on the growth of *Aureobasidium pullulans* strain Fito_F278. Responses of strain Fito_F278 to pH and salinity tolerance were recorded as follows: -, no growth; +, growth. Results are of two biological replicates for each condition and the experiment was carried out three times.

Further, strain Fito_F278 was able to grow under a gradient of pH ranging from 5 to 11 (Figure 3a) and no significant differences were found on strain abundance (CFU/mL) (data not shown). However, the morphology of colonies was slightly altered, becoming smaller from pH 9. Under salinity conditions, Fito_F278 was able to grow up to 8% NaCl (Figure 3b). Significant differences (*p* < 0.05) of strain abundance (CFU/mL) were found between standard conditions (0% NaCL) and 4, 6, and 8% NaCl, respectively (data not shown). In the meantime, the morphology of colonies was altered with NaCl, becoming smaller by increasing the NaCl concentration in the culture medium.

### *In vivo* Assays

#### Colonization of Grapevine by *Aureobasidium pullulans* Strain Fito_F278

**FIGURE 4:**
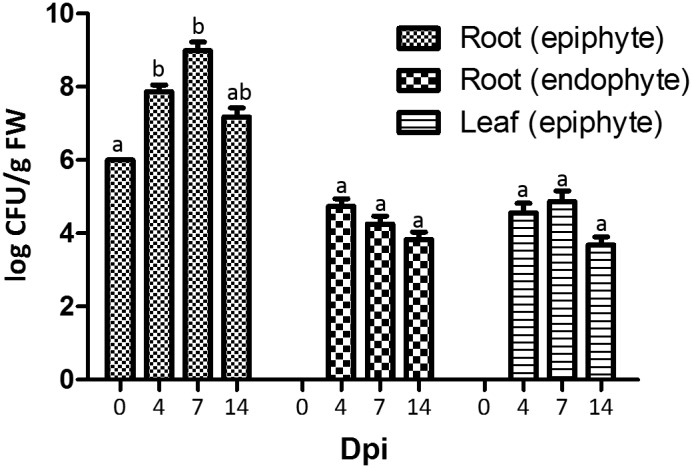
Colonization of plantlets of *Vitis vinifera* cv. Chardonnay by *Aureobasidium pullulans* strain Fito_F278 at 0, 4, 7 and 14 days post-inoculation (dpi). Two biological replicates (five pooled plantlets) from each condition and from two experiments are presented. The CFU count was log-transformed and the pooled mean and standard error values of 2 + 2 replicates from the two experiments are presented for each condition. Differences between time points within each plant tissue were compared to determine their significance. Letters indicate significant differences according to the Kruskal–Wallis test, followed by a Mann–Whitney *U* test (*p* ≤ 0.05). FW, fresh weight.

To address the plant colonization capacity of strain Fito_F278, roots of plantlets cv Chardonnay were dipped in a strain solution at 10^6^ CFU/mL and then allowed to grow under *in vitro* conditions, in a photoperiod chamber. At 4 dpi, the strain CFU count at root surfaces increased 74-fold (10^7^ CFU/g FW), when compared to 0 dpi (Figure 4). Furthermore, Fito_F278 was also detected at both internal root tissues (10^4^ CFU/g FW) and leaf surfaces (10^4^ CFU/g FW). At 7 dpi, the CFU count was similar for both internal root tissues and leaf surface, while a 13-fold higher levels were detected at root surfaces. However, such colonization decreases at 14 dpi. Interestingly, Fito_F278 was able to colonize the inner tissues of leaves even if in very low quantities and in a non-systematic way; in other words, Fito_F278 was not always present overtime. Despite the fluctuations on CFU count, no statistical differences were found overtime for each plant tissue in analysis.

Regarding the plant-Fito_F278 interaction, effects on both plant growth and development were observed in inoculated plantlets (Supplementary Figure S3). Thus, after a dense colony layer (biomass) formation on plant roots at 7 dpi (Supplementary Figures S2, S3), the plant growth was affected and both discoloration and spot necrosis on leaves were observed (Supplementary Figure S4a). At 14 dpi, plants showed short and dark roots (Supplementary Figure S4b). Contrary, the inoculation of strain Fito_F278 did not cause any visual symptoms in cutting plants (Supplementary Figure S5).

**FIGURE 5:**
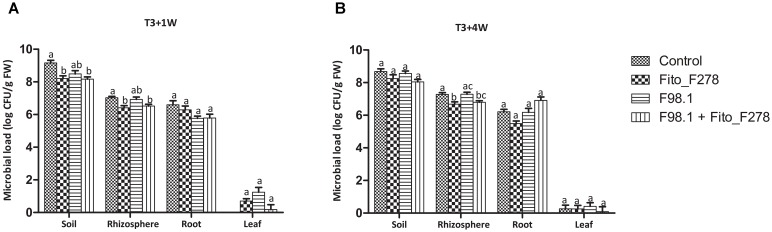
Analysis of the microbial load present in cutting plants at T3+1 week and T3+4 weeks. Results showed the bacterial and yeasts isolated from soil, rhizosphere, roots and leaves samples at T3+1 week **(A)** and T3+4 weeks **(B)**, through a classical microbiology analysis. Results from roots and leaves correspond to the endophytic population. Three biological replicates for each condition and time point and from two experiments are presented. The CFU count was log-transformed and the pooled mean and standard error values of 3 + 3 replicates from the two experiments are presented for each condition. Differences between treatments within each plant tissue were compared to determine their significance. Letters indicate significant differences according to the Kruskal–Wallis test, followed by a Mann–Whitney *U* test (*p* ≤ 0.05).

In terms of cutting colonization, other microorganisms than the strain Fito_F278 were isolated. As expected, the isolated bacteria and/or yeasts showed a higher CFU count in soils when compared with the other plant tissues (Figure 5). Furthermore, filamentous fungi were also isolated and a higher CFU count was obtained in soils (data not shown). Given the strain Fito_F278, this was detected in soils and inside roots, confirming its endophytic potential. In soils, it was able to survive up to 1 month after its inoculation (T3+1 week), and then the inner tissues of roots seemed to have constituted a favorable environment for its development under longer periods (T3+4 weeks). Strain Fito_F278 was not detected in control plants (non-inoculated plants). Interestingly, a significant decrease of the microbial load from soil and rhizosphere was observed in cuttings treated with the strain Fito_F278 (Figure 5). The use of strain-specific primers targeting the GST gene, and the sequencing of the ITS region allowed to identify the strain Fito_F278 from the other re-isolated microorganisms. The PCR amplification with strain-specific primers originated an expected band with 753 bp and the specificity tests showed that the selected primers did not amplify other *A. pullulans* strains and bacteria (Supplementary Figure S2).

#### Effect of *Aureobasidium pullulans* Strain Fito_F278 on Both Frequency and Necrotic Lesions Caused by Diplodia Seriata in Grapevine Cuttings

The green stems inoculated alone or co-inoculated with *D. seriata* F98.1 + Fito_F278 exhibited external necrotic lesions associated with the artificial inoculation of the phytopathogen at 1 month (T3+4 weeks) after its inoculation. These wood lesions were determined for their width and length and used for phytopathogen re-isolation analysis. As expected, *D. seriata* F98.1 was not recovered from lesions of control conditions and was re-isolated only from necrotic lesions of inoculated plants, thus ensuring the Koch’s postulate. Results showed that *D. seriata* F98.1 was present only at the artificial inoculation point (IP), and never upward or downward of IP.

**FIGURE 6:**
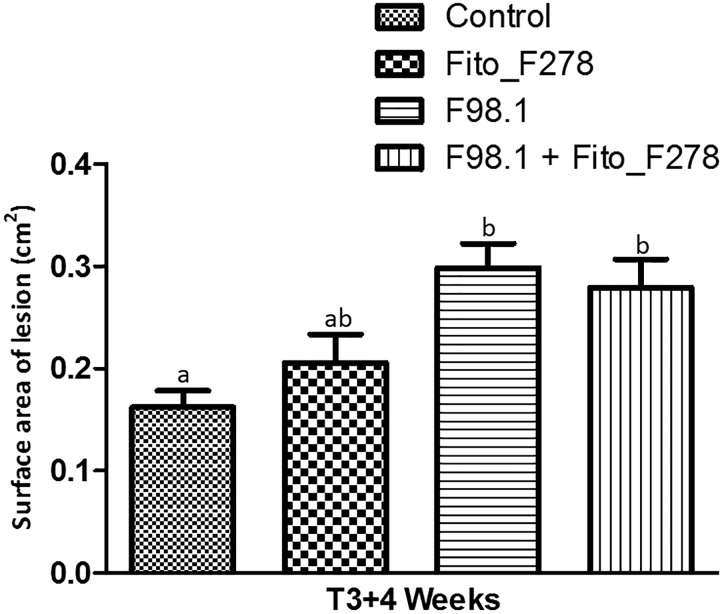
Surface area of necrotic lesion on green stems (cv. Chardonnay) after artificial inoculation. Results correspond to the surface area of necrotic lesion (cm^2^) on green stems measured 1 month (T3+4 weeks) after their artificial inoculation. Control and Fito_F278 conditions where inoculated with a sterile PDA plug, while F98.1 and F98.1 + Fito_F278 conditions where inoculated with *Diplodia seriata* F98.1. Seven replicates were assessed for each condition and the experiment was carried out twice. The pooled mean and standard error values of 7 + 7 replicates from two experiments are presented for each condition. Letters indicate significant differences according to the one-way ANOVA analysis [*F*(3,52) = 6.881; *p* = 0.01; adjusted *R*^2^= 0.243] followed by a Bonferroni *post hoc* test (*p* < 0.05).

When considered the relative frequency of *D. seriata* F98.1 recovered at IP, this was 10.4% lower when strain Fito_F278 was present (Table 2). Meanwhile, the surface area of lesions of green stems caused by the phytopathogen infection tended to be lower on plants inoculated with strain Fito_F278 (Figure 6). Overall, the surface area of lesions of green stems from plants inoculated with *D. seriata* F98.1 reached an average of 0.30 ± 0.02 cm^2^, while plants co-inoculated with phytopathogen + Fito_F278 showed an average of 0.28 ± 0.03 cm^2^.

**Table 2:** Number of isolates (n) and relative frequency (%) of *Diplodia seriata* F98.1 recovered from the inoculation point (IP) of cuttings cv. Chardonnay.

	T3+1 week		T3+4 weeks	
Conditions (total number of wood pieces)	Positive replicates (*n)*	Relative frequency (%)	Positive replicates (*n)*	Relative frequency (%)
F98.1 *(n* = 48)	6	12.50 ± 12.50^a^	20	41.67 ± 16.37^a^
F98.1 + Fito_F278 *(n* = 48)	4	8.33 ± 5.46^a^	15	31.25 ± 10.65^a^

*The re-isolation process was performed on the green stems from plants inoculated alone with F98.1 and co-inoculated with F98.1 + Fito_F278, at one (T3+1 week) and four (T3+4 weeks) weeks after the artificial inoculation of the phytopathogen. Results are based on 48 wood pieces of green stems from a total of eight plant replicates for each condition and time point. The pooled mean and standard error values of 4 + 4 plant replicates from two experiments are presented for each condition. Differences between treatments and time points were compared to determine their significance. Letters indicate significant differences according to the Kruskal–Wallis test, followed by a Mann–Whitney U test (p ≤ 0.05).*

#### Relative PSII Activity Analysis

Overall, the plant inoculation with strain Fito_F278 did not affect significantly the activity of PSII (Supplementary Figure S6). However, a significant non-systematic increase of PSII activity was observed for some time-points, namely at T3+3 days and T3+3 weeks in co-inoculated plants (F98.1 + Fito_F278).

#### Effect of *Aureobasidium pullulans* Strain Fito_F278 on Grapevine Defense Responses

In order to determine how grapevine responds to strain Fito_F278 in the presence or absence of *D. seriata* F98.1, a set of 8 genes were selected for analysis. These genes encoded for plant defense proteins (*PR6, Gluc*), detoxification and stress tolerance (*Hahl, HSP, GST5*), phenylpropanoid pathway (*STS*), cell wall (*fascAGP*), and water stress (*Pip 2.2*) were selected from previous studies focusing on grapevine responses to GTDs (Magnin-Robert et al., 2011; Reis et al., 2016; Spagnolo et al., 2017).

**FIGURE 7:**
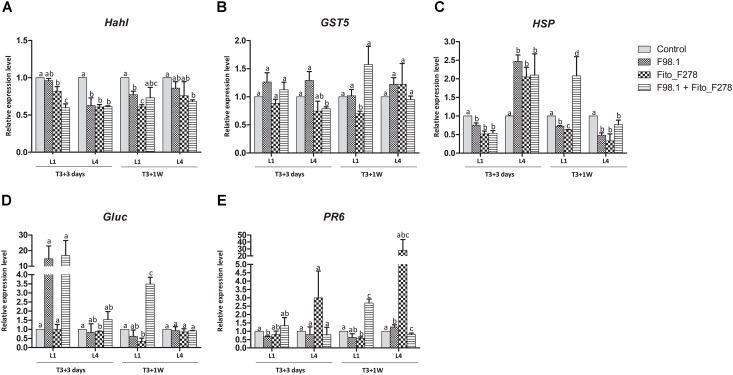
Relative expression level of 5 genes in leaves from plants inoculated alone or co-inoculated with *Aureobasidium pullulans* strain Fito_F278 and *Diplodia seriata* F98.1. The relative expression of genes encoding for detoxification and stress tolerance, such as haloacid dehalogenase hydrolase (*HahL*) **(A)**, glutathione S-transferase (*GST5*) **(B)** and α-crystalline heat shock protein (*HSP*) **(C)**, and for pathogenesis-related proteins, such as β-1,3 glucanase (*Gluc*) **(D)** and PR protein 6 (*PR6*) **(E)** was determined by qPCR at T3+3 days and T3+1 week after the artificial inoculation of phytopathogen. Both the first (L1) and the fourth (L4) leaves above the artificial inoculation spot of the phytopathogen were analyzed. Results represent the relative expression levels of reported conditions in relation to the control leaves (non-inoculated plants), defined with a relative expression of 1.0 and represented by the dotted line on the graphs. Two experiments each one with a pool of four biological replicates and two technical replicates (4 × 2 = 8) for each condition and time point are presented. The pooled mean and standard error values of 8 + 8 values from the two experiments are presented for each condition and time point. Differences between treatments within each leaf and time point were compared to determine their significance. Letters indicate significant differences according to the Kruskal–Wallis test, followed by a Mann–Whitney *U* test (*p* ≤ 0.05). The expression of a given gene was considered up- or down- regulated when changes in relative expression were > 2-fold or <0.5- fold, respectively. Only genes with significant differences from the control and a cut off above the threshold were considered as significantly modulated.

**FIGURE 8:**
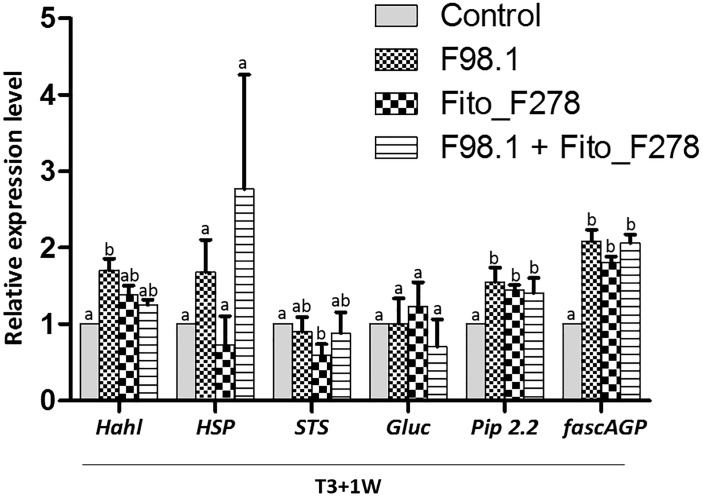
Relative expression level of 6 genes in green stems from plants inoculated alone or co-inoculated with *Aureobasidium pullulans* strain Fito_F278 and *Diplodia seriata* F98.1. The relative expression of genes encoding for detoxification and stress tolerance: haloacid dehalogenase hydrolase (*HahL*) and α-crystalline heat shock protein (*HSP*); phenylpropanoid pathway: stilbene synthase (*STS*); pathogenesis-related proteins: PR protein 6 (*PR6*); cell wall compound synthesis: fasciclin-like arabinogalactan protein (*fascAGP*); and aquaporin plasma membrane intrinsic protein 2-2 (*PIP2.2*) was determined by qPCR at T3+1 week after the artificial inoculation of phytopathogen. Results represent the relative expression levels of reported conditions in relation to the control green stems (non-inoculated plants), defined with a relative expression of 1.0 and represented by the dotted line on the graphs. Two experiments each one with a pool of four biological replicates and two technical replicates (4 × 2 = 8) for each condition and time point are presented. The pooled mean and standard error values of 8 + 8 values from the two experiments are presented for each condition and time point. Differences between treatments were compared to determine their significance. Letters indicate significant differences according to the Kruskal–Wallis test, followed by a Mann–Whitney *U* test (*p* ≤ 0.05). The expression of a given gene was considered up- or down- regulated when changes in relative expression were >2-fold or <0.5-fold, respectively. Only genes with significant differences from the control and a cut off above the threshold were considered as significantly modulated.

Results showed that some plant responses differed across the plant (L1 vs. L4 or leaf vs. green stem), sampling time (T3+3 days vs. T3+1 week) and treatment conditions (Figures 7, 8). Overall, transcripts such *Hahl* and GST5 were not affected in leaves though, *HSP, Gluc* and *PR6* trend to be overexpressed in co-inoculated plants: *Gluc* and *PR6* were up-regulated at L1 (T3+1 week), whereas *HSP* was up-regulated at both L1 (T3+1 week) and L4 (T3+ 3 days) (Figure 7). In green stems, transcripts such as *Hahl, HSP, STS, Gluc* and *Pip2.2* were not affected though, fascAGP were up-regulated in co-inoculated plants and with the pathogen alone (Figure 8).

Regarding plants artificially inoculated with the phytopathogen alone showed an up-regulation of *HSP* at T3+3 days (L4). However, such gene was not affected at T3+1 week. No changes were reported at green stems, though *fascAGP* was up-regulated. Grapevine responses of plants inoculated with strain Fito_F278 alone showed an up-regulation of *HSP* at T3+3 days (L4), followed by a down-regulation of both *HSP* and *Gluc* at T3+1 week (L4 and L1, respectively). Given the green stems, the relative expression level of genes was not affected for these plants.

## Discussion

Herein a deep characterization of *Aureobasidium pullulans* strain Fito_F278, a resident microorganism from grapevine microbiome (Grube et al., 2011; Pinto et al., 2014, 2015), was performed as no evidence of its antagonistic potential was reported against *D. seriata*, a Botryosphaeria dieback agent. For this reason, an analysis of *A. pullulans* strain Fito_F278 was carried out to limit this gap and to promote a better understanding of its interaction with grapevine and potential relevance for biocontrol of GTDs, under *in vitro* and *in planta* conditions.

### Efficient Endophytic Colonization of Plant Roots by the Strain Fito_F278

Grapevine plantlets were effectively colonized by the strain Fito_F278, namely from roots to leaves, at both epiphytic and endophytic levels, confirming its endophytic potential. In cuttings, such colonization occurred only for soils and for the internal tissues of roots. In the former, strain Fito_F278 persisted up to 1 month after its inoculation and in the later, seemed to have constituted a favorable environment for its development and survival under longer periods. The colonization of the internal tissues of leaves was observed but was not systematic. These results are in accordance with those of McGrath and Andrews (2007) in which the authors showed that the interveinal sites of apple leaves remained poorly colonized after a high inoculation with *A. pullulans*. Other studies have evidenced a widespread pattern of *A. pullulans* to colonize grapevine such as pruning wounds, wood, leaves, grapes and musts (Munkvold and Marois, 1993; Martini et al., 2009; Pinto et al., 2014, 2015; Fischer et al., 2016). In addition, *A. pullulans* can be fully found across different plants such as apple, cucumber, cabbage, cereal grains, food products and water (Deshpande et al., 1992; Vero et al., 2009).

Remarkably, and in contrast to grapevine cuttings, the growth and fitness of plantlets were affected by the presence of strain Fito_F278 after 7 dpi. The high strain inoculum, together with the high nutrient availability of the culture media [sucrose content at 3% (w/v)] should be the causes of such results since it leads to a rapid plant colonization that may have triggered some plant defense mechanisms, causing the plant symptoms emergence. Furthermore, the development of a dense cell colony layer (biomass) over the top layer of culture media and around plant roots may be related to the production of some extracellular polysaccharides (EPS). Several studies showed the importance of culture medium in the production of EPS. Among them, a study of Singh et al. (2012) showed that an optimal concentration of sucrose at 3% (w/v) in a fermentation batch and at 42°C resulted in a higher production of EPS, namely pullulan when compared with other carbon sources such as fructose, glucose, lactose or xylose. Furthermore, it was considered that the pullulan production can be stimulated by the addition of certain doses of ammonium sulfate or ammonium nitrate (Ravella et al., 2010). In fact, this step showed to have play a role in the increasing of the biofilm’s quantity produced by *A. pullulans* and, which consequently increased its antagonistic activity against *Geotrichum citri-aurantii*, the causal agent of sour rot in citrus (Klein and Kupper, 2018). Considering that in our study, the culture media used to grow the grapevine plantlets contained a sucrose content of 3% (w/v) and ammonium nitrate, this may undoubtedly have contributed to the production of some EPS as most likely pullulan.

### Some Mechanisms of Action of *Aureobasidium pullulans* May Explain Its Ubiquitous Colonization Over Different Plants

Some of the potential mechanisms of action used by *A. pullulans* strain Fito_F278 during the direct antagonistic activity and plant colonization were investigated. Data from dual cultures support evidences of a competition for both space and nutrients of Fito_F278 against the phytopathogen, constituting one potential mode of action used by this strain for its antagonistic response. Furthermore, our results showed that strain Fito_F278 produced hydrolytic enzymes recognized for its biotechnological potential such as amylase, cellulase, lipase, pectinase and proteinase, under *in vitro* conditions, which is in agreement with other previous studies (Deshpande et al., 1992). Interestingly, strain Fito_F278 has a highest enzymatic index for cellulase and pectinase. Considering that cellulose and pectin are components of the plant cell-wall, the production of cell-wall degrading enzymes seems to pose a threat to plant. However, it is recognized that the synthesis of such enzymes is in some cases a strategy adopted by yeasts, namely *A. pullulans*, and other endophytes to obtain carbon sources and, thus to ensure their capacity to adapt and to successful colonize the inner tissues of plants (Biely and Kremnický, 1998; Compant et al., 2005b; Reinhold-Hurek and Hurek, 2011; Liu et al., 2017). Indeed, the genome analysis of four *A. pullulans* varieties (Gostinčar et al., 2014) or even other endophytic bacteria of grasses (Straub et al., 2013) and rice (Sessitsch et al., 2012) revealed several genes encoding cell-wall degrading enzymes.

In addition, strain Fito_F278 produced siderophores, solubilized the phosphate, could grow at different pH and is resistant to high salinity conditions. Meanwhile, the morphological forms of *A. pullulans* colonies were affected by pH and salinity conditions which agree with other morphological studies (Deshpande et al., 1992; Gaur et al., 2010). From a biocontrol point of view, the pH and salinity conditions have no direct relevance however, these traits express the capacity of Fito_F278 to survive under harsh environmental conditions. Moreover, pH, temperature and nutrient sources (carbon or nitrogen) have an important role on different products biosynthesis such as the EPS pullulan or glucan, through a stimulation or a suppression (Gaur et al., 2010; Singh et al., 2012). The ability of *A. pullulans* to produce several hydrolytic enzymes, and its tolerance to salt concentrations and pH has already been reported (Deshpande et al., 1992; Buzzini and Martini, 2002; Zalar et al., 2008). Likewise, *A. pullulans* is a copper and sulfur tolerant microorganism (Grube et al., 2011; Schmid et al., 2011; Pinto et al., 2014), which ensures its prevalence in vineyards. Its tolerance to metal ions, pollutant compounds of soils and water, is also recognized (Gaur et al., 2010). The phenotypic plasticity of *A. pullulans* promotes certainly its tolerance to different ecological conditions, thus allowing the colonization of several niches and guaranteeing its adaptability and survival.

Finally, our results showed a significant decrease of the microbial load from soil and rhizosphere of grapevine cutting treated with strain Fito_F278. Considering that *A. pullulans* is an ubiquitous and indigenous microorganism from grapevine, this may be related with its competitivity potential against other microbial communities, in order to improve and to ensure its survival.

#### Monitoring the Plant Colonization by Strain Fito_F278 Using Strain-Specific Primers

Strain-specific primers of *A. pullulans* strain Fito_F278 were developed to monitor and to easily identify this strain across grapevine tissues. Despite ITS region is the most commonly used for species identification (White et al., 1990), this is a conserved region among species and does not confine an intra-species distinction. Thus, the Glutathione S-Transferase C (GST) gene, mainly involved in the detoxification process and tolerance to oxidative stress (Sheehan et al., 2001; McGoldrick et al., 2005), was here used since it allowed an intra-species discrimination among *A. pullulans* strains. Other specific primers for *A. pullulans* targeting the ITS2 region were also developed by Martini et al. (2009) to detect endophytic colonization of these microorganisms on grapevine leaves and shoots. However, and as ITS2 region is conserved across *A. pullulans* strains, these primers only allowed an inter-species identification. Schena et al. (2002) also analyzed the genetic variability of different *A. pullulans* strains by RAPD and synthetized a sequence-characterized amplified region (SCAR) primers. Other household genes are described in literature for phylogenetic analysis of *A. pullulans*, encoding proteins such as actin (ACT), β-tubulin (BTUB), translation elongation factor 1α (EF1α), calmodulin (CAL), elongase (ELO), NAD-dependent glycerol-phosphate dehydrogenase (GPD) or RNA polymerase 2 largest subunit (RPB2) (Zalar et al., 2008; Gostinčar et al., 2014).

Herein, a molecular analysis together with a viable cell count allowed an accurate analysis of strain Fito_F278 colonization. Therefore, molecular methods are more sensitive and faster than CFU analysis. Considering that *A. pullulans* is an ubiquitous and highly abundant microorganism, these strain-specific primers will be clearly useful for a rapid identification of strain Fito_F278 on greenhouse and field experiments.

### *Aureobasidium pullulans* Strain Fito_F278 Showed a Direct Antagonistic Activity Against Diplodia Seriata F98.1

In our study, *A. pullulans* strain Fito_F278 significantly reduced the mycelium growth of *D. seriata*, via direct antagonism and under *in vitro* conditions. The highest levels of antagonistic activity were observed for *D. seriata* strain F98.1 with a mycelium inhibition of 33.41 ± 0.55%. Nevertheless, *D. seriata* strain Ds99.7, which is characterized as the highest aggressive strain, was the less susceptible to the mycelium inhibition (7.98 ± 0.95%).

Despite the efficacy of strain Fito_F278 under *in vitro* conditions, no significant reduction of disease lesions and relative frequency were found in cutting plants, reinforcing that the antagonistic activities of this strain are dependent on a direct interaction with the phytopathogen. Thus, further studies using direct application treatments rather than applications at soil level are therefore required.

To date, and to the best of our knowledge, there is only one study available that applied *A. pullulans*, isolated from pruning wounds, to control *Eutypa lata*, a GTD agent (Munkvold and Marois, 1993). In this study, two field experiments were performed in California region: the first one in 1990 in a Thompson Seedless vineyard, and the second one in 1991 in a cv. Chenin Blanc vineyard. A set of natural occurring microorganisms was applied in cane-pruned to test their efficacy as BCA. Among them, *A. pullulans* significantly reduced the infection, with a reduction superior to 50% compared to control treatment, only in the first field. Another study revealed that *Aureobasidium* isolates from cv. Chardonnay inhibited the growth of *Greeneria uvicola*, a bitter rot agent of bunches, by dual antagonistic tests, detached berries, leaves, and bunches of potted grapevines (Rathnayake et al., 2018). In addition, *A. pullulans* is mainly reported as an important BCA of an abroad of post-harvest diseases such as in apple fruit (*B. cinerea* and *Penicillium expansum*), cherry tomato and kiwifruit (*B. cinerea*), sweet cherry (*B. cinerea* and *Monilia laxa*), strawberries, and table grape (*B. cinerea, P. expansum, Rhizopus stolonifera* and *Aspergillus niger*) (Ippolito et al., 2000; Castoria et al., 2001; Schena et al., 2002; Bencheqroun et al., 2007; Vero et al., 2009). Other studies revealed that *A. pullulans* reduced the *Fusarium* head blight (FHB), a devastating disease of common wheat caused mainly by *Fusarium culmorum*, with a decrease in disease severity of 21.67% (Wachowska and Glowacka, 2014). Overall, these observations reinforce that grapevine hosts an ubiquitous microorganism with an antagonistic potential, that may constitute a primary physic barrier against phytopathogens, and emphasize the opportunity to use *A. pullulans* as a BCA. However, the success of its biocontrol potential must be achieved by optimizing its functioning in agroecosystems (Toju et al., 2018).

### The Strain Fito_F278 Activated Some Plant Defense Responses in Presence of the Phytopathogen

In order to better understand the grapevine responses to strain Fito_F278 in the presence or absence of *D. seriata*, certain gene activities were analyzed. Globally, no major modifications were reported in plants only inoculated with strain Fito_F278. These results suggest that strain Fito_F278, as an ubiquitous and endophytic microorganism, might be not perceived as an invader by the plant and, therefore, justify its potential biocontrol use.

Interestingly, plant responses changed in the co-inoculated conditions (phytopathogen + Fito_F278) mostly at T3+1 week. Herein, the expression of targeted genes involved in the detoxification and stress tolerance (*HSP*) and defense proteins (*Gluc* and *PR6*) were overexpressed in leaves, namely at L1. In green stems, no modifications occurred, whereas *fascAGP* remained up-regulated. The significant induction of PR protein gene encoding for glucanases (*Gluc)* 1 week after the inoculation of *D. seriata* F98.1, represent a plant resistance strategy to limit the spread of the phytopathogen. Proteins produced by these genes have an important role in breaking-down the cell wall components of phytopathogens (Funnell et al., 2004).

Similar trends were described by Haidar et al. (2016) where pre-treated plants with BCA did not induce a plant response after the phytopathogen *P. chlamydospora* inoculation. Likewise, a down-regulation of defense-related genes was also noted in plants co-inoculated with the BCA *P. fluorescens* (PTA-CT2) and the pathogen *B. cinerea* (Gruau et al., 2015).

## Conclusion

To conclude, this study reports a certain novelty measure since, and to the best of our knowledge, this is the first report on the interaction of *A. pullulans* with grapevine and its potential role against *D. seriata*, a Botryosphaeria dieback agent. Main findings are that *A. pullulans* strain Fito_F278 has an antagonistic effect against *D. seriata* through a direct interaction, and induces some plant defense responses. Moreover, the endophytic potential of strain Fito_F278, its capacity to persist on plant roots for long-term and its capacity to grow at different pH and high salinity conditions reinforce its competitive advantages useful in further vineyard applications.

Considering that *A. pullulans* is a naturally colonizer of grapevine, these findings underline the hypothesis that *A. pullulans* may have a natural role on the competition with agents of GTDs in the field. However, new direct application treatments, different inoculum concentrations, and a deep analysis of the interaction of strain Fito_F278 with other non-target microorganisms should be considered in the future.

## Author Contributions

CP conducted the design of experiment, experimental work, and writing of the manuscript. VC was associated with the microbial isolation from gapevine and molecular characterization. MN was associated with the physico-chemical characterization of Fito_F278 strain. AS was associated with the *in vivo* assays for colonization and biocontrol activity assessment. FR helped for molecular analysis and validation. BC prepared the *in vitro* plants of grapevine for this study. CC reviewed the manuscript and contributed with consumables. AG and FF supervised, coordinated the experiments, contributed with consumables, wrote and critically revised the manuscript.

## Conflict of Interest Statement

The authors declare that the research was conducted in the absence of any commercial or financial relationships that could be construed as a potential conflict of interest.
