# Supplementary material for: Understand the Potential Role of Aureobasidium pullulans, a Resident Microorganism From Grapevine, to Prevent the Infection Caused by Diplodia seriata
Source: Front Microbiol. 2018 Dec 11;9:3047. doi: 10.3389/fmicb.2018.03047 (PMC6297368; doi:10.3389/fmicb.2018.03047)
Supplement: Supplementary file 2 [file Data_Sheet_1.PDF]

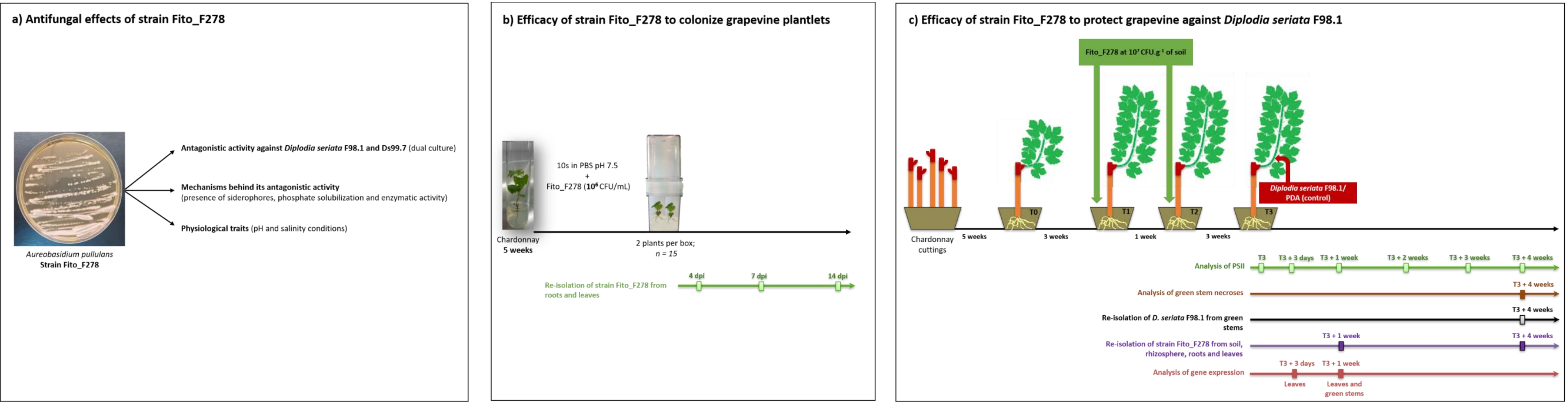

Figure S1: General scheme of the experimental design representing all the approaches performed under this study. The characterization of *Aureobasidium pullulans* strain Fito_F278 was achieved for its antifungal effects (a), efficacy to colonize grapevine plantlets (b), and protection of grapevine against *Diplodia seriata* F98.1 (c). dpi: days post-inoculation.
