# Supplementary material for: Understand the Potential Role of Aureobasidium pullulans, a Resident Microorganism From Grapevine, to Prevent the Infection Caused by Diplodia seriata
Source: Front Microbiol. 2018 Dec 11;9:3047. doi: 10.3389/fmicb.2018.03047 (PMC6297368; doi:10.3389/fmicb.2018.03047)
Supplement: Supplementary file 3 [file Data_Sheet_2.PDF]

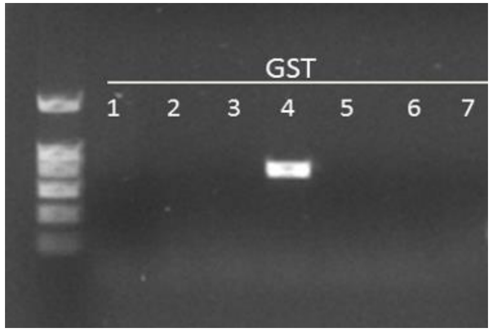

Figure S2: Strain-specific primers validation. DNA from different *A. pullulans* strains and bacteria was loaded with the strain-specific primers targeting the Glutathione S-transferase C (GST) gene. The PCR amplification originated an expected band with 753 bp amplicon. M: 200 bp DNA ladder (NZYDNA Ladder I, Nzytech); 1: *A. pullulans* strain Mean 522; 2: *A. pullulans* strain Fito_F261; 3: *A. pullulans* strain Fito_F312; 4: *A. pullulans* strain Fito_F278 (C+ - positive control); 5: *Bacillus amyloliquefaciens* subsp. *plantarum* strain Fito_F321 (MG836692); 6: *Streptomyces* sp. strain Fito_S127B; 7: C- (negative control - sterile water instead of DNA).
