# Supplementary material for: Understand the Potential Role of Aureobasidium pullulans, a Resident Microorganism From Grapevine, to Prevent the Infection Caused by Diplodia seriata
Source: Front Microbiol. 2018 Dec 11;9:3047. doi: 10.3389/fmicb.2018.03047 (PMC6297368; doi:10.3389/fmicb.2018.03047)
Supplement: Supplementary file 4 [file Data_Sheet_3.PDF]

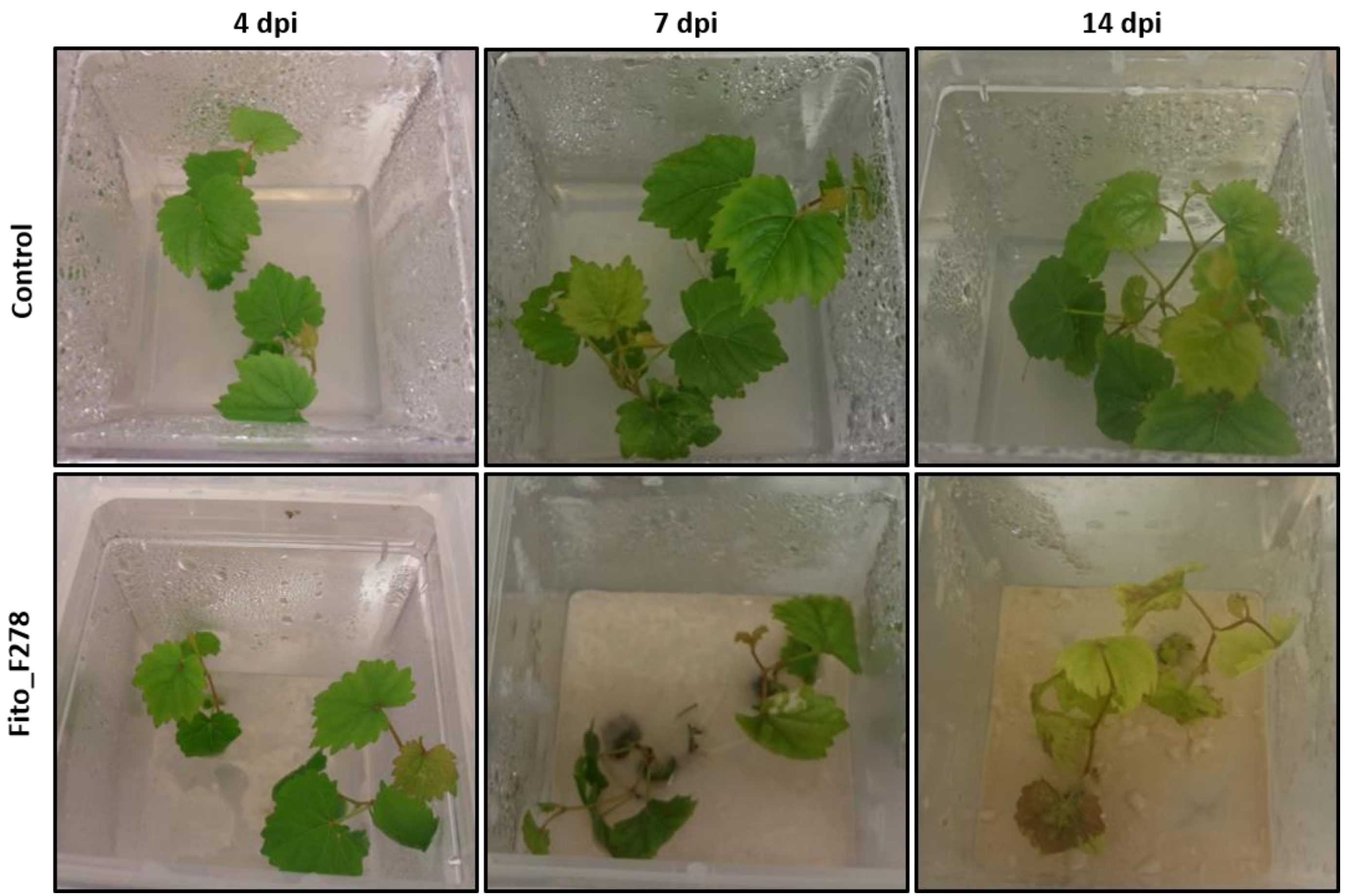

Figure S3: Effect of the *Aureobasidium pullulans* strain Fito_F278 inoculation on *Vitis vinifera* cv. Chardonnay performance. Control plants (non-inoculated) and plantlets inoculated with strain Fito_F278 have been analysed at 4, 7 and 14 days post-inoculation (dpi). Results from one representative experiment (out of two) was selected to produce the figure.
