# Supplementary material for: Understand the Potential Role of Aureobasidium pullulans, a Resident Microorganism From Grapevine, to Prevent the Infection Caused by Diplodia seriata
Source: Front Microbiol. 2018 Dec 11;9:3047. doi: 10.3389/fmicb.2018.03047 (PMC6297368; doi:10.3389/fmicb.2018.03047)
Supplement: Supplementary file 5 [file Data_Sheet_4.PDF]

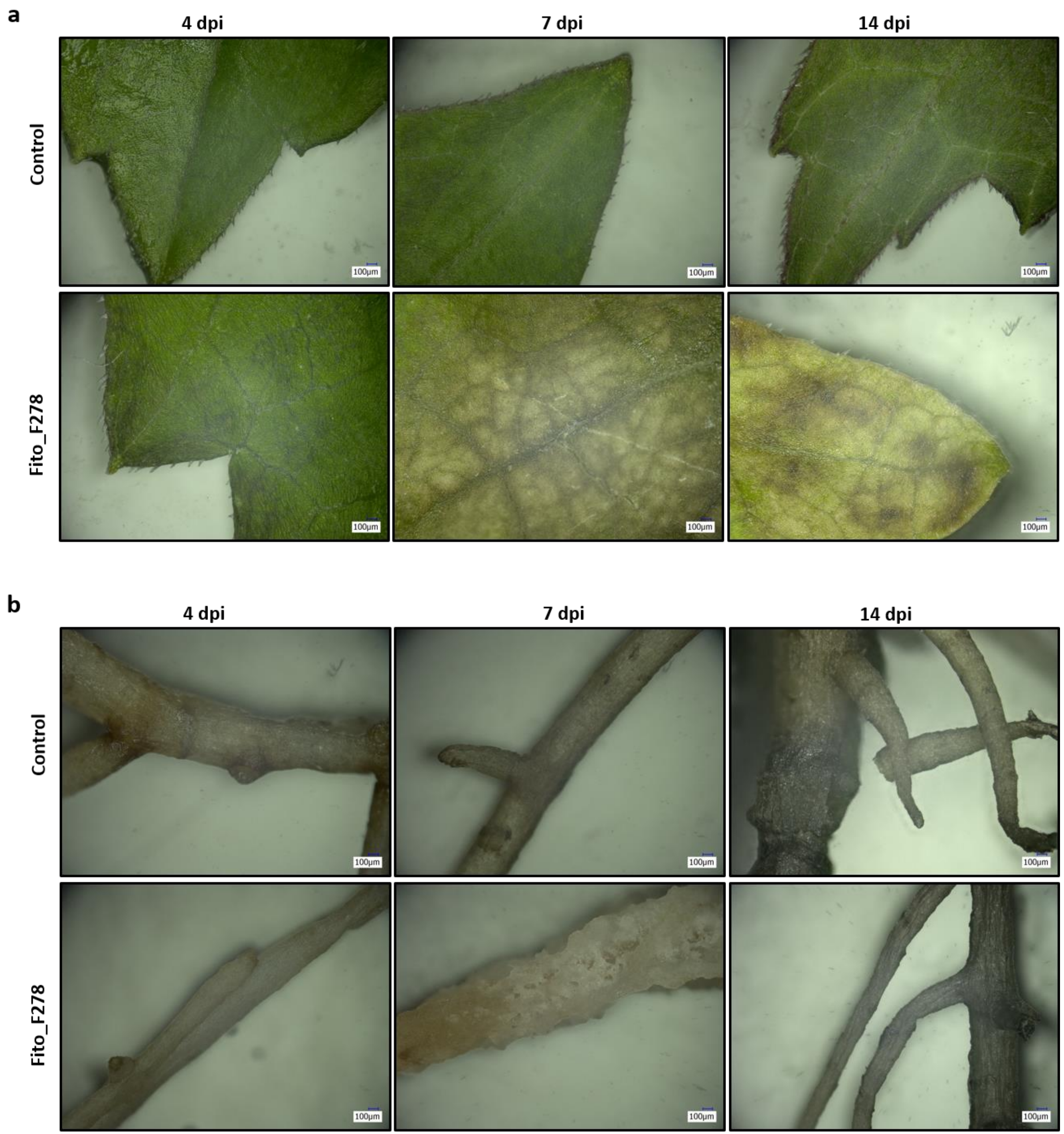

Figure S4: Morphological traits of *Vitis vinifera* cv. Chardonnay non-inoculated (control) and inoculated with strain Fito_F278. 3D-microscopy analysis of leaves (a) and roots (b) at 4, 7 and 14 days post-inoculation (dpi). Bars = 100 µm. Results from one representative experiment (out of two) was selected to produce the figure.
