# Supplementary material for: Understand the Potential Role of Aureobasidium pullulans, a Resident Microorganism From Grapevine, to Prevent the Infection Caused by Diplodia seriata
Source: Front Microbiol. 2018 Dec 11;9:3047. doi: 10.3389/fmicb.2018.03047 (PMC6297368; doi:10.3389/fmicb.2018.03047)
Supplement: Supplementary file 6 [file Data_Sheet_5.PDF]

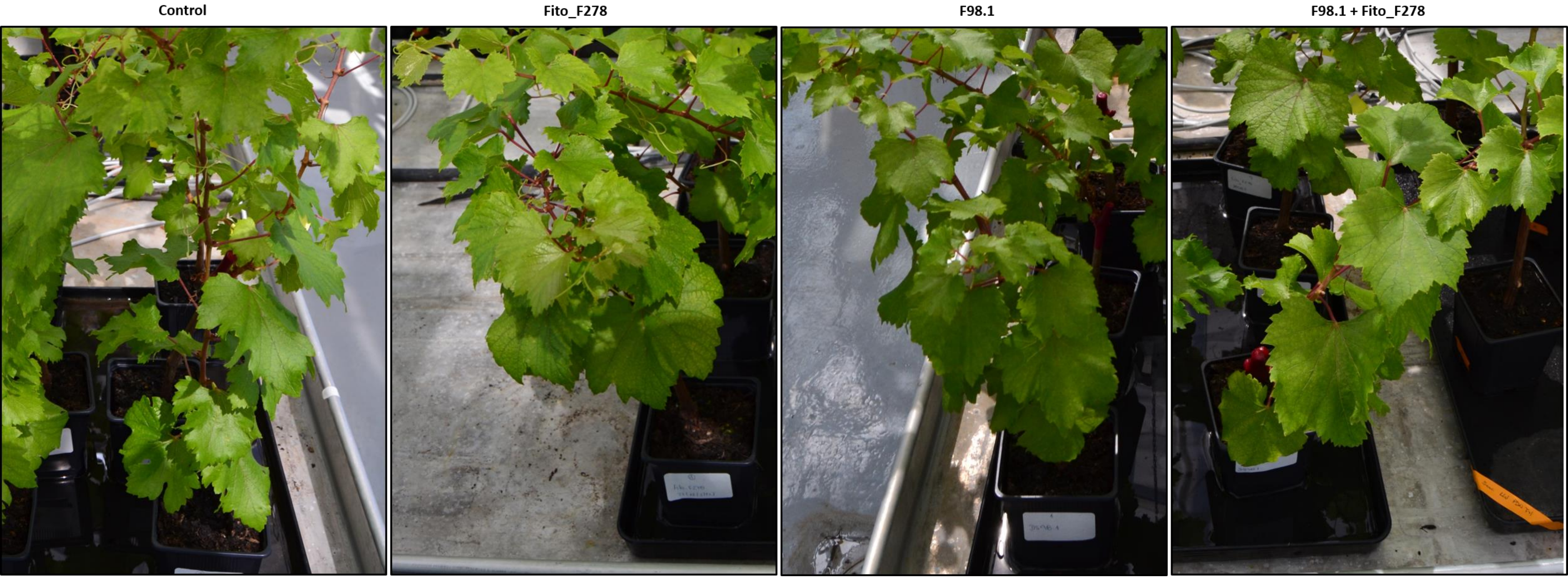

Figure S5: Effect of the *Aureobasidium pullulans* strain Fito_F278 inoculation on *Vitis vinifera* cv. Chardonnay performance. Results showed the control plants (non-inoculated), cuttings inoculated with strain Fito_F278, *D. seriata* F98.1 and both F98.1 + Fito_F278 at T3+4 weeks. Results from one representative experiment (out of two) was selected to produce the figure.
