# Supplementary material for: Understand the Potential Role of Aureobasidium pullulans, a Resident Microorganism From Grapevine, to Prevent the Infection Caused by Diplodia seriata
Source: Front Microbiol. 2018 Dec 11;9:3047. doi: 10.3389/fmicb.2018.03047 (PMC6297368; doi:10.3389/fmicb.2018.03047)
Supplement: Supplementary file 7 [file Data_Sheet_6.PDF]

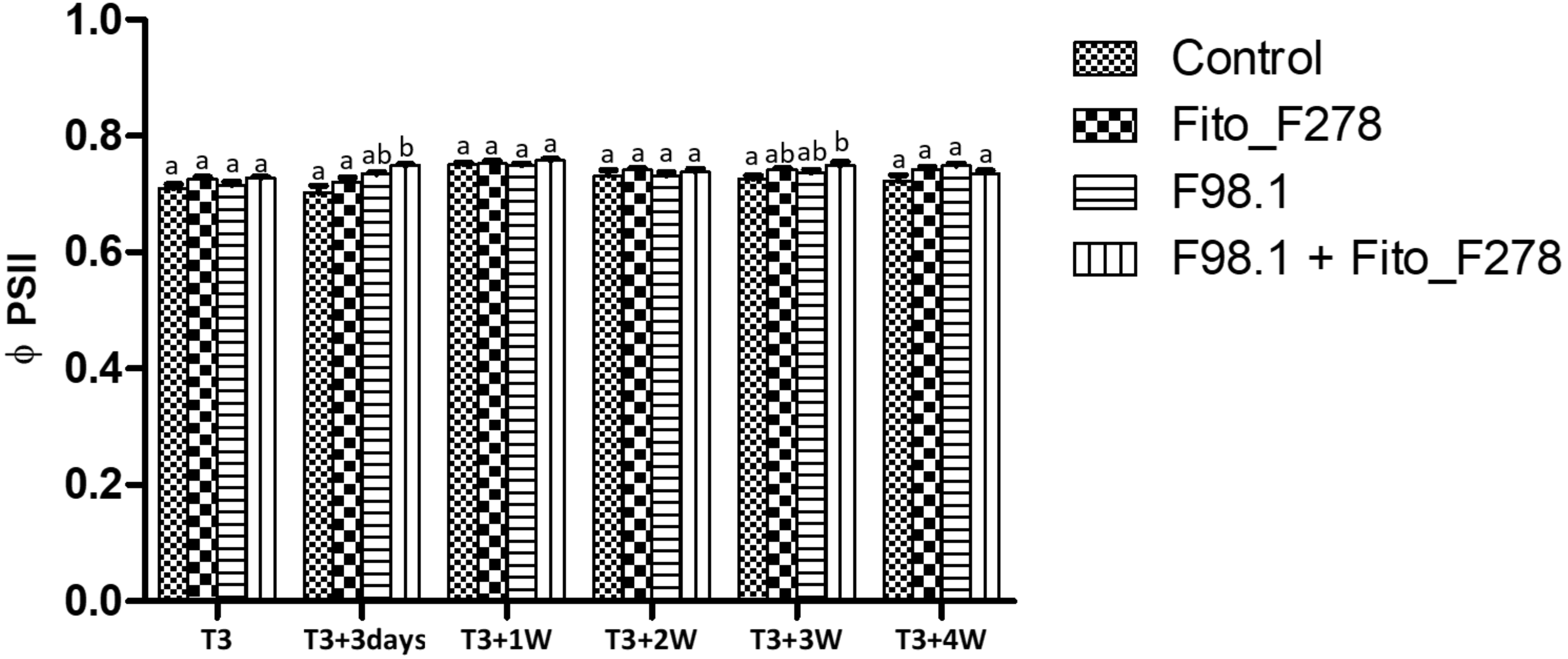

Figure S6: Changes in relative quantum yield of photosystem II (φPSII) overtime. Results of 11 (T3; T3+3 days; T3+1 week) and 7 replicates (T3+2 weeks; T3+3 weeks; T3+4 weeks) were assessed for each condition and the experiment was carried out twice. The pooled mean and standard error values of replicates from the two experiments are presented for each condition and time point. Differences between conditions within each time point were compared to determine their significance. Letters indicate significant differences according to the Kruskal–Wallis test, followed by a Mann–Whitney U test ($p \leq 0.05$).
